# A study on smartphone dependence and depression in Korean high school students

**DOI:** 10.1097/MD.0000000000033354

**Published:** 2023-03-24

**Authors:** Soohee Park, Jin-Yeong Yoo

**Affiliations:** a Department of Occupational Therapy, Honam University, Gwangsan-gu, Gwangju-si, Gyeonggi-do, South Korea; b Division of Rehabilitation, Suseong University, Suseong-gu, Daegu, South Korea.

**Keywords:** depression, dependence on smartphone, Korean high school student, smartphone, internet addiction

## Abstract

Using the 10th Korean Youth Risk Behavior Web-based Survey in 2020, we attempted to determine the relationship between smartphone addiction and depression in 25,987 high school students. The demographic characteristics of the subjects were reviewed frequently, and the correlation between smartphone dependence and depression were determined. The results show that it is difficult to track the time spent on smartphones to determine the level of depression (*R* = 0.143, *P* < .01), it is difficult concentrating on other tasks due to smartphones (*R* = 0.140, *P* < .01), and it is difficult to remove images/data received on smartphones from the head (*R* = 0.141, *P* < .01). Further, the impulse to use smartphones is strong (*R* = 0.157, *P* < .01), health concerns (r = .124, *P* < .01) and family conflicts exist due to the use of smartphones (r = .149, *P* < .01), problems with social relations exist due to the use of smartphones (*R* = 112, *P* < .01), and difficulties exist in performing tasks due to the use of smartphones (*R* = 0.153, *P* < .01). According to the results of the study, it was determined that there is a relationship between smartphone usage and depression among Korean high school students. The findings from this study can be referenced to help guide the development of smartphone usage parameters for Korean teenagers in depression management programs.

## 1. Introduction

Adolescence is a period of various psychosocial difficulties, such as many changes in life cycle development, stress and confusion, and emotional impulsivity.^[1]^ Today, teenagers and those born after the year 2000, known as the digital media generation, and have essentially been exposed to the digital environment since birth.^[2]^ In 2018, the rate of smartphone use for high school students in Korea was 95.2%, and most teenagers had smartphones. For teenagers, the internet is an important environmental factor affecting socialization; it is also a key force in social change that creates an internet-based culture unique to teenagers.^[3]^ In particular, due to the recent coronavirus disease 2019 pandemic, the dependence on smartphones has been increasing.^[4]^ The dependence on smartphones reduces the ability to control smartphone use. Smartphones limit daily life and important activities.^[5]^ Indeed, overuse of smartphones and excessive dependence on smartphones are emerging as addiction problems.^[6]^

Depression increases after school age.^[7]^ For adolescents, the ability to self-regulate is underdeveloped, and adolescent depression caused by excessive smartphone use is an avoidable phenomenon experienced in adolescence.^[8]^ Adolescent depression is not only an issue of self-awareness, but parents also often fail to recognize the existence of depression among their children.^[9]^ Adolescents depression causes problematic behaviors such as substance abuse and Internet addiction. Also, depression affects Internet game addiction.^[10]^ During adolescent development the areas of psychological, emotional, and social areas are highly susceptible to external environmental factors that can increase vulnerability to addiction. The external environment factor is influenced by the home environment, school life, and friendship.^[11]^ The proportion of Korean adolescents’ dependence on smartphones was 30.3% in 2017.^[12]^

Depression is a complex mental health issue that affects a significant proportion of Korean high school students. While there have been efforts to prevent and manage depression among teenagers, the causes and management of depression remain insufficiently addressed. Additionally, the increasing dependence on smartphones among Korean high school students has become a major social concern, which may be associated with depression. Therefore, the present study aims to investigate the relationship between smartphone dependence and depression among Korean high school students in a more rigorous manner. Specifically, we hypothesize that there is a significant positive correlation between smartphone dependence and depression among Korean high school students. By providing a better understanding of this relationship, this study will offer insights for developing effective measures to prevent and manage depression among Korean high school students and promote a more healthy and sustainable use of smartphones.

## 2. Materials and methods

### 2.1. Research design and data collection

This descriptive study on the relationship between smartphone dependence and depression among Korean high school students utilized the 16th (2020) Korean youth risk behavior web-based survey (KYRBWS) conducted by the Centers for Disease Control and Prevention (CDC) at their headquarters. A total of 57,912 students from 800 schools (400 middle schools and 400 high schools) participated in the survey, with a participation rate of 94.9% from 793 schools and 54,948 students. From this data, 25,987 students (13,523 males and 12,464 females) were selected for analysis in order to investigate the relationship between smartphone use and depression among high school students. This study was approved by the government as a statistical survey (Approval No. 117058), and the data collection process followed the stratified village extraction method, where sample schools were selected based on factors such as geographical proximity, population, living environment, and number of schools, and one class was randomly selected from each grade of the secondary extraction unit. The ethical considerations of the Korean CDC’s original data disclosure and management regulations were followed, and permission was obtained prior to data collection.

### 2.2. Data collection

The tenth KYRBWS is conducted annually by the Ministry of Education, the Ministry of Health and Welfare, and the CDC.^[13]^This study was a government-approved statistical survey (Approval No. 117058). The 16^th^ (2020) KYRBWS was conducted on middle and high school students nationwide, as of April 2020, and samples were collected using the stratified village extraction method. The first extraction unit listed schools based on geographical proximity, number of schools, population, living environment, etc, and selected sample schools by systematic extraction. One class was randomly selected for each grade of the secondary extraction unit.

### 2.3. Ethical considerations

Informed consent was obtained from all participants prior to data collection, and the instrument and process used in the survey were approved by the institutional review board at Korea Centers for Disease Control and Prevention. We strictly adhered to the ethical guidelines for human research, including protection of privacy and confidentiality.

To ensure ethical use of the raw data, we followed the original data disclosure and management regulations of the Korean CDC, and utilized the 16 ^th^ (2020) KYRBWS forms for data collection and analysis. The original data request form was submitted to the website of the KYRBWS (https://www.kdca.go.kr/yhs/), and we obtained the information with permission from the Korea Centers for Disease Control and Prevention after the data utilization protocol was reviewed and approved (Approval No. 117058). We maintained the confidentiality of the data and ensured that it was used only for the purposes stated in the study protocol.

### 2.4. Selection of research variables

For research purposes, depression mitigation rates and smartphone usage items were used.

#### 2.4.1. Depression experience rate.

This variable was used “How happy do you usually feel?.” Depression was rated on a (Likert) scale of 1 to 5, where 1 indicated happiness, 2 indicated, moderately less happiness, 4 indicated almost no happiness, and 5 indicated unhappiness.

#### 2.4.2. Dependence on smartphones.

The results of 8 out of the 10 smartphone dependency screening tools were published. The selection tool items included the following: It is difficult to maintain the right time to use smartphones; It is hard to concentrate on other things when you have a smartphone; I cannot get my smartphone out of my head; I strongly felt the urge to use smartphones; Smartphone use can cause health problems; I had a heated argument about using a smartphone with my family; I have experienced severe conflicts with my friends, colleagues, and social relationships due to smartphone use, and Smartphones make it difficult to carry out work (study, occupation, etc). The 8 items of the screening tool were used with each criteria containing a 4-point rating scale. For each assessment, total points were calculated, and higher scores were linked to a higher dependence on smartphones.

### 2.5. Data analysis

In this study, the demographic characteristics of the participants were analyzed frequently. To verify the relationship between dependence on smartphones and depression-related variables, this study analyzed the Pearson correlation with statistical significance set at *P* < .05. SPSS (21.0, IBM, Armonk, NY) was used to perform all analyses.

## 3. Results

### 3.1. Demographic characteristics

Of all the children included, 34.3% were in the 10th grade, 34.3% in the 11th grade, and 31.5% in the 12th grade; 79.3% were from ordinary high schools, 19.1% were from specialized high schools, and 1.5% were from “other” high schools. The characteristics of residential areas were as follows: 43.8 % metropolitan areas, 47.7% small and medium-sized cities, and 8.6% from rural areas (Table 1).

**Table 1:** Demographic characteristics (N = 25,987).

Variable		Number (%)
Sex	Male	13,523 (52.0)
	Female	12,464 (48.0)
Grade	10	8907 (34.3)
	11	8907 (34.3)
	12	8173 (31.5)
School type	General high school	20,616 (79.3)
	Specialized high school	4976 (19.1)
	Others	395 (1.5)
City type	Metropolitan	11,374 (43.8)
	Mid-sized cities	12,838 (47.7)
	Rural	2230 (8.6)

### 3.2. The correlation between adolescents depression and dependence on smartphones

The correlation and orientation between the depression experience and smartphone dependence, as the main variables of this study, are discussed. Two thousand seven hundred-fifty-seven 2757(10.6%) of the participants answered they were depressed. Pearson correlation analysis was performed. The results show that it is difficult to track the time spent on smartphones to determine the level of depression (*R* = 0.143, *P* < .01), it is difficult concentrating on other tasks due to smartphones (*R* = 0.140, *P* < .01), and it is difficult to remove images/data received on smartphones from the head (*R* = 0.141, *P* < .01). Further, the impulse to use smartphones is strong (*R* = 0.157, *P* < .01), health concerns (r = .124, *P* < .01) and family conflicts exist due to the use of smartphones (r = .149, *P* < .01), problems with social relations exist due to the use of smartphones (*R* = 112, *P* < .01), and difficulties exist in performing tasks due to the use of smartphones (*R* = 0.153, *P* < .01)(Table 2).

**Table 2:** Analysis of the correlation between depression and smartphone dependence (N = 25,987).

Variable	1	2	3	4	5	6	7	8	
Experience of depression	1								
Difficulty tracking the time spent on smartphones	.143*	1							
Difficulty concentrating on other things	.140*	.658*	1						
Cannot get the information received from a smartphone out of one’s head	.141*	.591*	.622*	1					
Having a strong urge to use smartphones	.157*	.645*	.645*	.759*	1				
Smartphone-related health concerns	.124*	.393*	.373*	.505*	.473*	1			
Smartphone-related family conflicts	.149*	.401*	.390*	.459*	.466*	.498*	1		
Problems in social relations due to smartphones	.112*	.274*	.271*	.486*	.404*	.575*	.538*	1	
Difficulty in performing tasks	.153*	.573*	.605*	.558*	.584*	.474*	.464*	.419*	1

* *P* < .01.

## 4. Discussion

This study was conducted to investigate the correlation between depression-related behaviors and smartphone addiction among Korean high school students, based on the 16th KYRBWS. The correlation and orientation between the depression experience and smartphone dependence, as the main variables of this study, are discussed. The rate of Korean students using smartphones is 95%, ranking Korea number one in the world, and it is an estimated 20% higher rate than other countries.^[14]^ According to the 16th KYRBWS, it was reported that 25.2 % of the students felt sad and desperate enough to stop their daily lives for 2 weeks over the past 12 months. Among them, 30.7% were female students and 20.1% were male students. Within the same group, it was determined that 25 percent of the students were at risk of smartphone use or dependence, with 30.3% being women and 21.2% being men. The research hypothesis was established based on the results of previous studies that Korean high school students dependence on smartphones causes daily stress and psychological maladjustment.^[15]^ Based on the purpose of the study, Korean high school students dependence on smartphones and depression was investigated, and findings indicate a static correlation between variables.

In other words, it is difficult to concentrate on other things because it can be tough to keep track of time when on a smartphone. Excessive internet use can seriously damage academic, professional, and psychological factors, which is commonly a feature used to diagnose addiction disorders.^[16]^ The results suggest that the more you think about smartphones, the higher the depression, but adolescents who are inexperienced in coping with stress express various forms of problematic behavior and social malpractice,^[10]^ which appears to manifest as dependence on smartphones.

In Korea, teenagers are generally discouraged from using smartphones; however, depression and interpersonal problems have led teenagers to rely on smartphones as a coping mechanism.^[17]^ The strong urge to use smartphones proves the correlation between difficulty in controlling impulsive use and depression. Although smartphone-related health problems and depression also have a static correlation, studies have shown that they have a significant impact on mental health.^[18]^ Family conflicts over smartphones are also intensifying, but teenagers regard smartphones as a medium to meet their needs.^[19]^ The age of smartphone addiction seems to be getting younger, as evidenced by recent studies. In their systematic review and meta-analysis, Ko and colleagues found that programs aimed at addressing smartphone addiction in Korean youth were effective in improving not only smartphone addiction, but also related factors such as anxiety, depression, impulsiveness, self-control, and self-esteem.^[20]^ Smartphone use can cause problems in social relationships, because smartphones can lead to poor regulation and problematic consequences this can lead to social contraction.^[21]^ Research has also shown that smartphone use has a negative impact on business engagement.^[22]^ Although there are many studies on the effects of each variable, there is not enough research on the relationship between variables and depression, and the results of this study reveal that all variables investigated are related to depression.

The limitations of this study include the fact that the sample may not be representative of all Korean high school students, as the number of people surveyed is not large. Furthermore, since this study is a secondary analysis, there is a limit to the selection of variables. Existing statistical methods and prior research can predict results, but there is a limit to the implementation of additional controls, other than those identified by the investigation.^[23]^

Research has shown that chronic fatigue and depression are experienced by senior students of Dicle University, which may be a useful reference to further evaluate the discussion section.^[24]^ Additionally, previous studies have shown that smartphone dependence can have an effect on depression, which supports the findings of this study.^[25]^ For example, Yilmaz et al^[26]^ (2016) found that somatosensory amplification, anxiety, and depression in patients with hepatitis B had an impact on functionality. Despite these limitations, this study is significant in that it uses national statistical data to collect representative high school students from all over the country and uses large-scale research data. The results of this study provide basic information that can prevent dependence on smartphones and depression rates in young people, as soon as possible by arbitrating related lawyers. The findings suggest that all items related to smartphone dependence are related to depression and that practical strategies are needed to address this issue.^[23]^

Therefore, the purpose is to find practical strategies and provide basic information that can prevent dependence on smart phones and depression rates in young people, as soon as possible by arbitrating related lawyers. Studies have shown that all items related to smartphone dependence are related to depression. Some studies have shown that smartphone dependence has an effect on depression. Although Korea is an technology powerhouse, research on the relationship between smartphone use and high school students depression is still insufficient, and the number of people surveyed is not large, therefore it cannot be seen as a representative sample of all high school students. This study is significant in that it uses national statistical data to collect representative high school students from all over the country and uses large-scale research data.

This study is a cross-sectional study using a youth health morphology survey, which can provide information on the correlation between depression and smartphone dependence. However, depression incidence over time, dependence on smartphones, and causal relationships between various factors cannot be clarified. In addition, the frequency of depression may differ from the actual frequency of depression because it is based on a self-report questionnaire. Furthermore, since this study is a secondary analysis, there is a limit to the selection of variables. Existing statistical methods and prior research can predict results, but there is a limit to the implementation of additional controls, other than those identified by the investigation.

## 5. Conclusions

This study investigated the relationship between smartphone dependence and depression among Korean high school students and used the 2020 tenth Korean youth risk behavior web-based survey as an assessment. The results showed a static correlation between smartphones, dependent variables, and depression. Therefore, in order to manage the depression of young people in Korea, education on smartphone use is necessary, and education programs should be developed at schools and homes to ensure systematic and correct smartphone use.

## Acknowledgments

I would like to thank the CHS investigator as well as Editage for English language editing.

## Author contributions

**Conceptualization:** Soohee Park, Jin-Yeong Yoo.

**Funding acquisition:** Soohee Park.

**Investigation:** Soohee Park.

**Supervision:** Jin-Yeong Yoo.

**Writing – original draft:** Soohee Park.

**Writing – review & editing:** Jin-Yeong Yoo.
